# Preparation and Characterization of Solution-Processed Nanocrystalline *p*-Type CuAlO_2_ Thin-Film Transistors

**DOI:** 10.1186/s11671-018-2680-5

**Published:** 2018-08-30

**Authors:** Shuang Li, Xinan Zhang, Penglin Zhang, Xianwen Sun, Haiwu Zheng, Weifeng Zhang

**Affiliations:** School of Physics and Electronics, Key Laboratory of Photovoltaic Materials, Henan University, Kaifeng, 475004 People’s Republic of China

**Keywords:** Solution-processed, *p*-type thin-film transistor, Nanocrystalline films, Mobility

## Abstract

The development of *p*-type metal oxide thin-film transistors (TFTs) is far behind the *n*-type counterparts. Here, *p*-type CuAlO_2_ thin films were deposited by spin coating and annealed in nitrogen atmosphere at different temperature. The effect of post-annealing temperature on the microstructure, chemical compositions, morphology, and optical properties of the thin films was investigated systematically. The phase conversion from a mixture of CuAl_2_O_4_ and CuO to nanocrystalline CuAlO_2_ was achieved when annealing temperature was higher than 900 °C, as well as the transmittance, optical energy band gap, grain size, and surface roughness of the films increase with the increase of annealing temperature. Next, bottom-gate *p*-type TFTs with CuAlO_2_ channel layer were fabricated on SiO_2_/Si substrate. It was found that the TFT performance was strongly dependent on the physical properties and the chemical composition of channel layer. The optimized nanocrystalline CuAlO_2_ TFT exhibits a threshold voltage of − 1.3 V, a mobility of ~ 0.1 cm^2^ V^−1^ s^−1^, and a current on/off ratio of ~ 10^3^. This report on solution-processed *p*-type CuAlO_2_ TFTs represents a significant progress towards low-cost complementary metal oxide semiconductor logic circuits.

## Background

Over the past decades, metal oxide thin-film transistors (TFTs) have been extensively investigated for the next-generation active-matrix liquid crystal displays, organic light-emitting diode displays, and other emerging electronic circuits applications due to their excellent electrical properties and outstanding optical transparency [1, 2]. However, a majority of the metal oxide TFTs reported to date were focused on *n*-type materials [3]. The *p*-type oxide semiconductors are usually characterized with localized oxygen *2p* orbitals with large electronegativity, self-compensation from oxygen vacancies, and the incorporation of hydrogen as an unintentional donor. Therefore, it is difficult in achieving effective hole doping [4]. Up to now, only a few *p*-type oxide materials (Cu_2_O, CuO, SnO, etc.) were proved to be suitable for TFT application [5, 6], but their performance lags far behind of *n*-type counterparts. This limits the development of all oxide *p-n* junctions and complementary metal oxide semiconductor (CMOS) logic circuits.

To obtain a good *p*-type metal oxide, it is critical to modify the energy band structure and reduce the Coulomb force exerted by the oxygen ions on holes. thus motivating the discovery of a group of *p*-type delafossite oxides, such as CuMO_2_ (M=Al, Ga, In) and SrCu_2_O_2_ [7, 8]. Among them, CuAlO_2_ has a wide bandgap of ~ 3.5 eV, and its valence band maximums are dominated by a large hybridization of the oxygen orbitals with 3d^10^ electrons in the Cu^1+^ closed shell, which leads to a dispersive valence band. Meanwhile, the cations with closed shells (d^10^s^0^) are beneficial to achieve high optical transparency because such an electronic structure can avoid light absorption from the so-called d-d transitions. Therefore, it has attracted considerable attention since the first fabrication in 1997 [9]. However, there are only a few reports focusing on *p*-type TFTs using CuAlO_2_ as channel layers. The primary difficulty is poor crystallinity and impurity phases, such as Cu_2_O, CuO, Al_2_O_3_, and CuAl_2_O_4_. The first report of a CuAlO_2_ TFT was fabricated by magnetron sputtering, and the device exhibits a current on/off ratio of 8 × 10^2^ and a hole mobility of 0.97 cm^2^ V^−1^ s^−1^ [10]. However, magnetron sputtering needs a strict high vacuum environment and a sophisticated operation process. In contrast, the solution-processed method offers conspicuous advantages, such as simplicity, low-cost, tunable composition, and atmospheric processing. In this work, we present a solution route to prepare CuAlO_2_ thin films. The effect of annealing temperature on the microstructure, chemical compositions, morphology, and optical properties of the thin films was investigated systematically. Finally, bottom-gate TFTs using the obtained nanocrystalline CuAlO_2_ thin films as channel layers were fabricated and they exhibit a mobility of ~ 0.1 cm^2^V^−1^ s^−1^, a threshold voltage of − 1.3 V, and a current on/off ratio of ~ 10 [3].

## Methods/Experimental

### Precursor Preparation and Thin-Film Fabrication

The CuAlO_2_ thin films were prepared by spin coating using copper nitrate trihydrate (Cu(NO_3_)_2_·3H_2_O) and aluminum nitrate nonahydrate (Al(NO_3_)_3_·9H2O) as starting materials. The molar ratio of two metal salts is 1:1, and the concentration of each salt in ethylene glycol methylether is 0.2 mol/L; acetylacetone was added to form a stable solution in deep green. The entire mixing process was carried out in 80 °C water bath with stirring. Prior to film deposition, the substrates were ultrasonically cleaned with acetone, ethanol, and deionized water for 5 min in each solution. Then, the final precursor was spin-coated with a low rotating speed of 500 rpm for 9 s and followed by a high rotating speed of 5000 rpm for 30 s. After spin-coating, the substrate was annealed at 350 °C for 20 min. The procedures from coating to annealing were repeated four times until the desired thickness (~ 40 nm) of the films was reached. Finally, the as-deposited films were annealed at 700–1000 °C for 2 h in nitrogen atmosphere and cooled down to room temperature at same atmosphere.

### Fabrication of CuAlO_2_ TFTs

The CuAlO_2_ TFTs with a bottom-gate structure were fabricated on SiO_2_/Si substrate. Three hundred-nanometer-thick SiO_2_ serves as the gate dielectric. After CuAlO_2_ film deposition, 50 nm gold source/drain electrodes were thermal evaporated on the channel layer through a shadow mask. The evaporation rate was 0.08 nm/s, and the channel width (W) and length (L) were 1000 μm and 100 μm, respectively. Finally, an indium layer was welded to the Si substrate as the back-gate electrode.

### Film and TFT Characterization

The CuAlO_2_ film structure was studied by X-ray diffraction (XRD, DX2500) with CuKα radiation (*λ* = 0.154 nm). The Raman spectrum was measured by Renishaw-1000 with a solid-state laser (633 nm). Surface morphologies were measured with scanning electron microscopy (SEM, JSM-5600LV, JEOL) and Veeco Dimension Icon atomic force microscopy (AFM). X-ray photoelectron spectroscopy (XPS) was performed on a Thermo Scientific Escalab 250 Xi spectrometer. XPS spectra were collected after etching the film surface for about 3 nm to minimize surface contamination. The optical transmittance was measured by UV-Vis spectrophotometer (Varian Cary 5000). The electrical characteristics were measured by a semiconductor parameter analyzer (Keithley 2612B).

## Results and Discussion

**Fig. 1:**
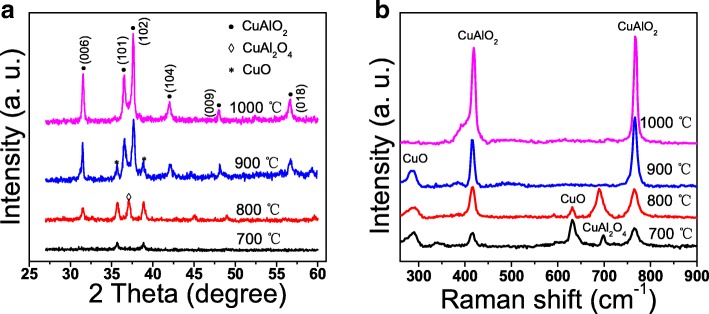
**a** XRD patterns. **b** Raman spectra of the CuAlO_2_ thin films annealed at different temperature

Figure 1a shows the XRD patterns of CuAlO_2_ thin films annealed at a different temperature. For the film annealed at 700 °C, only weak CuO phase diffraction peaks at 35.8° and 38.9° were observed, indicating that 700 °C is not enough for the formation of CuAlO_2_ phase [11]. Two new peaks at 31.7° and 37.1° assigned to CuAlO_2_ and CuAl_2_O_4_ phases, respectively, were observed after 800 °C annealing. When the temperature reached to 900 °C, the intensity of the CuO peaks decrease and the CuAl_2_O_4_ phase peaks disappear. Several new peaks at 36.8°, 42.5°, 48.5°, 57.5°, and 31.7°, assigned to CuAlO_2_ phase, dominated the film [9, 12]. The temperature was further increased to 1000 °C, the peaks intensity increased, and single CuAlO_2_ phase were obtained. The enhancement of the crystallinity may be attributed to the fact that more energy absorption accelerated the growth of crystallites at higher annealing temperature.

Figure 1b shows the Raman spectra of CuAlO_2_ thin films. The four-atom primitive cell of delafossite structure CuAlO_2_ leads to 12 normal modes, but only the A_1g_ (416 cm^−1^) and E_g_ (771 cm^−1^) modes are Raman active. As shown in Fig. 1b, it is obvious that two Raman vibration modes, A_1g_ and E_g_, both are present for all films [13]. Unlike bulk analysis of XRD, Raman scattering originates from the molecular vibration and lattice vibration that can detect Raman active molecule vibrate from a very small amount of concentration. That explains the existence of CuAlO_2_ phase in the 700 °C annealed film, which is not observable in XRD spectrum. Others peaks at 798 cm^−1^, 297 cm^−1^, and 632 cm^−1^ were also observed which are assigned to the F_2g_ mode of CuAl_2_O_4_, A_g_, and B_g_ modes of CuO, respectively [14]. The peaks of CuO and CuAl_2_O_4_ phases decrease as the annealing temperature increases from 700 to 1000 °C, and both phases convert to phase CuAlO_2_ after 1000 °C annealing, which is consistent with XRD results.

**Fig. 2:**
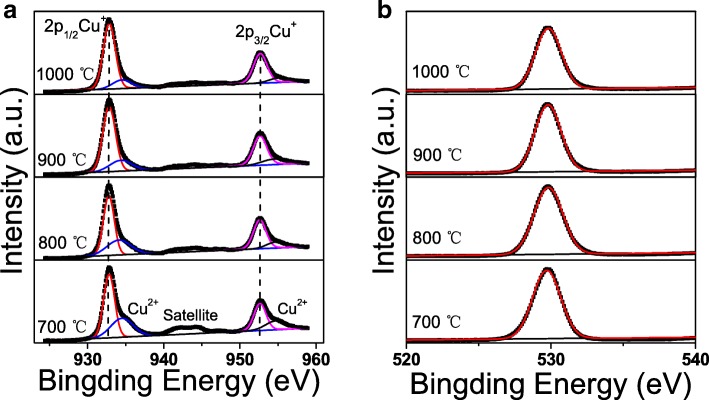
**a** Cu 2p. **b** O 1s XPS spectra of the CuAlO_2_ thin films annealed at different temperature

To understand the chemical compositions of CuAlO_2_ thin films annealed at different temperature, XPS measurement was carried out and the Cu 2p core levels spectra are shown in Fig. 2a. The typical Cu 2p3/2 peak can be fitted into two peaks located at ~ 932.8 and ~ 934.2 eV, which can be attributed to Cu^+^ and Cu^2+^, respectively. Similarly, fitted Cu 2p1/2 deconvolution two peaks are centered at ~ 952.6 (Cu^+^) and ~ 954.1 eV (Cu^2+^), respectively [15]. Since the spin-orbital splitting of Cu 2p is ~ 19.8 eV, the 2p3/2 and 2p1/2 peaks were not constrained for the area ratio during fitting. Nevertheless, the area ratio of the Cu 2p3/2 peak and the Cu 2p1/2 peak is ~ 1.90, being close to the ideal value of 2 determined from electron state densities [14]. The dominated peaks at ~ 932.8 eV (Cu^+^) and ~ 952.6 eV (Cu^+^) indicate that Cu cations mainly exist in Cu^+^ form in CuAlO_2_ lattice. Note that Cu^2+^ state is presented in all films, even no CuO peaks were detected in the high temperature annealed samples by XRD. Meanwhile, the satellite peaks observed from 941.2 to 944.4 eV also implied the presence of CuO. However, satellite peaks are almost negligible after high-temperature annealing, which is consistent with the above XRD observations. Quantitative analysis of the XPS spectra gave Cu^+^/[Cu^+^+Cu^2+^] atomic ratios of 62.5%, 68.9%, 73.7%, and 78.9% for CuAlO_2_ thin films annealed at 700, 800, 900, and 1000 °C, respectively, indicating reduction of Cu^2+^ with the increase of annealing temperature [10, 16]. The XPS O 1s peaks are shown in Fig. 2b. It was interesting that the binding energies exhibit symmetrical dominant peaks centered at ~ 529.8 eV, indicating most of oxygen atom are bonded to nearest neighbor metal ions (Cu^+^, Al^3+^, or Cu^2+^) in the lattice. It should be noted that the peak at ~ 531.3 eV, attributed to oxygen-related defects, can hardly be distinguished. This result can be explained by the introduction of surface etch process and high crystallization quality.

**Fig. 3:**
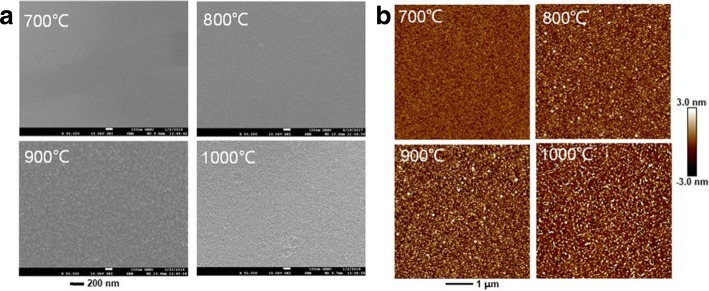
**a** SEM. **b** AFM of the CuAlO_2_ thin films annealed at different temperature

The surface morphologies of the CuAlO_2_ thin films were observed by SEM, as shown in Fig. 3a. All the films exhibit continuous, smooth, and dense structure morphology without obvious micro-cracks. The grain size is homogeneous and increases with the increase of annealing temperature. The gradually enlarged grain size would lead to the fewer grain boundaries, which act as trapping sites and significantly reduce the mobility for the nanocrystalline CuAlO_2_ films [17]. Thus, the CuAlO_2_ thin films annealing at high temperature is beneficial to the charge transport and may result high performance TFTs [18]. Surface roughness is another factor which can seriously influence the electrical performance of oxide TFTs [19]. To obtain the root mean square (RMS) roughness, the CuAlO_2_ thin films were investigated by AFM, as shown in Fig. 3b. The RMS roughness of films annealed at 700 °C, 800 °C, 900 °C, and 1000 °C were 0.92, 1.82, 2.12, and 2.96 nm, respectively. Obviously, the RMS increases with the increase of annealing temperature. Generally, rough surface would result in presence of electrical defects or trapping sites resulting in inferior device performance [20]. Therefore, it was supposed that there should be a competition relation between grain size and surface roughness to affect the performance of nanocrystalline oxide TFTs.

**Fig. 4:**
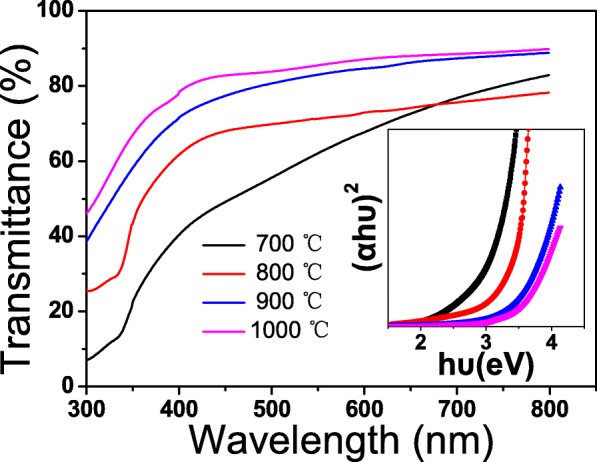
The optical transmission spectra for CuAlO_2_ films annealed at different temperature. The inset shows a plot of (*α*hν)^2^ vs. hν of the films

The optical transmission spectra of the CuAlO_2_ thin films on fused silica were measured in wavelength range from 200 to 800 nm, as shown in Fig. 4. It was observed that all films have steep absorption edge and strong ultraviolet absorption, which is an indication of the good crystallinity of the films. The average transmittance in the visible light region was calculated from ~ 60 to ~ 80%, increasing with the increase of annealing temperature. Tauc’s relation *αhν* = *A*(*h*ν − *E*_*g*_)^1/2^ is carried out to calculate the optical band gap, where *α* is the absorption coefficient, *A* is the constant for a direct transition, *h* is the Planck’s constant, and *ν* is the photon frequency [21]. The value of *Eg* is given by the linear extrapolation of the plot of *(αhν)*^*2*^ versus *hν* to the energy axis, as shown in the inset of Fig. 4. The *E*_*g*_ were calculated to be 3.25 eV, 3.40 eV, 3.60 eV, and 3.80 eV for the CuAlO_2_ thin films annealed at 700 °C, 800 °C, 900 °C, and 1000 °C, respectively.

**Fig. 5:**
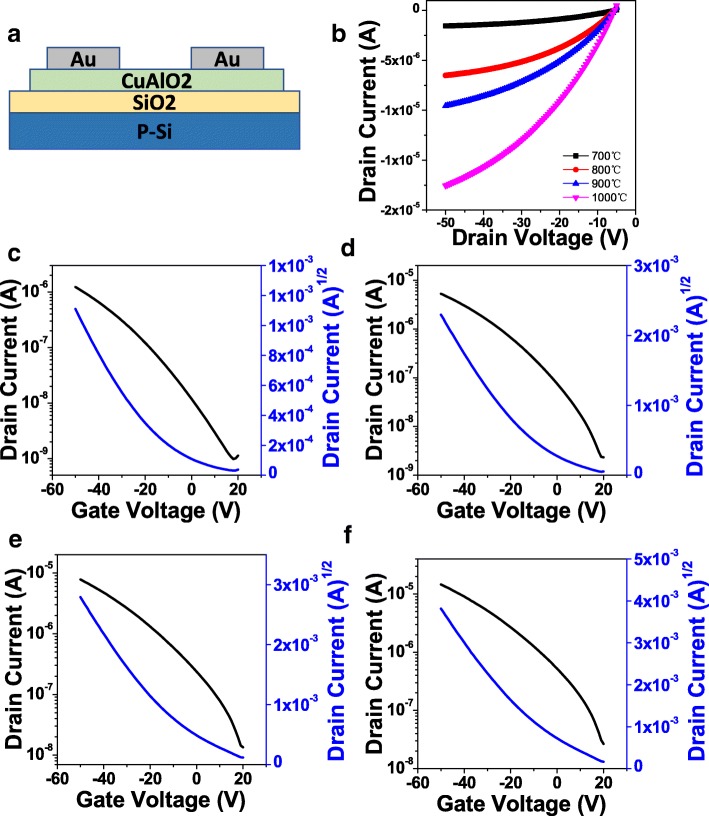
**a** Schematic diagram of the CuAlO_2_ TFTs. **b** Summarized output curves. **c**–**f** Transfer curves of the CuAlO_2_ TFTs annealed at 700 °C, 800 °C, 900 °C, and 1000 °C, respectively

Finally, we fabricated the bottom-gate top-contact TFTs on SiO_2_/p-Si substrates to investigate the electrical performance of CuAlO_2_ as channel layers. The schematic diagram of device is illustrated in Fig. 5a. The output curves of the CuAlO_2_ TFTs at a gate-source voltage (*V*_GS_) of − 50 V are shown in Fig. 5b. It clearly indicates the on-state current (*I*_on_) increases with the increase of annealing temperature. This is mainly attributed to the elimination of insulator-like CuAl_2_O_4_ phase and the enhancement of nanocrystalline CuAlO_2_ phase. The transfer curves are shown in Fig. 5c–f, and the CuAlO_2_ TFTs exhibit a typical *p*-type behavior. All the devices show a moderate on/off current ratio (*I*_on_/*I*_off_) of ~ 10^3^ which perhaps can be further improved by optimizing channel thickness, cation doping, or changing source/drain material [22–24]. The threshold voltage (*V*_T_) is determined as a horizontal axis intercept of a linear fitting to the *I*_DS_^1/2^-*V*_GS_ curve. The *V*_*T*_ shift towards the positive with the increase of annealing temperature. The filed effect mobility (*μ*_FE_), the subthreshold slope (SS), and the interface trap density (*N*_t_) can be calculated by the following equations [25, 26]:

1 $$ {I}_{\mathrm{DS}}=\frac{1}{2}{\mu}_{\mathrm{FE}}{C}_{\mathrm{OX}}\frac{W}{L}{\left({V}_{\mathrm{GS}}-{V}_{\mathrm{T}}\right)}^2 $$

2 $$ \mathrm{SS}={\left(\frac{d\left({\log}_{10}{I}_{\mathrm{DS}}\right)}{d{V}_{\mathrm{GS}}}\right)}^{-1} $$

3 $$ {N}_{\mathrm{t}}=\left[\frac{\mathrm{SSlog}(e)}{kT/q}-1\right]\left(\frac{C_{\mathrm{ox}}}{q}\right) $$

where *k* is the Boltzmann constant, *T* is the temperature, *q* is the elementary charge of electron, and *C*_ox_ is the areal capacitance of the gate insulator [27]. The key electrical parameters of the devices are listed in Table 1. It can be seen the SS values, largely higher than reported *n*-type devices, decrease with the increase of annealing temperature, which is consistent with the trend of *V*_T_. The results can be explained by the reduction of traps at the channel/dielectric interface [28]. The *μ*_FE_ values increase from 0.006 to 0.098 cm^2^V^−1^ s^−1^ as the annealing temperature increased from 700 to 1000 °C, which indicates an improvement of hole transport due to phase conversion from a mixture to nanocrystalline CuAlO_2_ and enlargement of grain size. The *μ*_FE_ are lower than solution-processed CuCrO_2_ TFTs reported by Nie et al [16]. The reason may be the delafossite nanocrystalline CuAlO_2_ structure is lack of Cu-O-Cu lattice content than that of CuCrO_2_ [29]. In spite of the annealing temperature of the devices is high for practical applications, this is the first report about solution-processed CuAlO_2_ TFTs. Further reduction of the annealing temperature by UV/ozone photochemical reaction and/or combustion synthesis is now underway [23, 30, 31].

**Table 1:** Electrical parameters of CuAlO_2_ TFTs with different annealing temperature

Annealing temperature (°C)	*I*_on_ (A)	Mobility (cm^2^ V^−1^ s^−1^)	*I*_on_/*I*_off_	*V*_T_ (V)	SS (V)	*N*_t_ (10–17 cm^−2^)
700	1.23 × 10^−6^	0.006 ± 0.004	~ 103	− 14.7 ± 0.8	14.9	1.79
800	5.27 × 10^−6^	0.028 ± 0.008	~ 103	− 9.5 ± 0.6	9.1	1.10
900	7.79 × 10^−6^	0.051 ± 0.011	~ 103	− 2.9 ± 0.5	9.0	1.07
1000	1.45 × 10^−5^	0.098 ± 0.009	~ 103	− 1.3 ± 0.5	8.6	1.03

## Conclusions

In summary, solution-processed CuAlO_2_ thin films were fabricated and annealed in nitrogen atmosphere at different temperature. With the temperature increased from 700 to 1000 °C, the film structure phase transforms from a mixture of CuAl_2_O_4_ and CuO to nanocrystalline CuAlO_2_, as well as the optical transmittance, energy band gap, grain size, and surface roughness of the films increase. The *p*-type CuAlO_2_ TFTs performance was strongly dependent on the physical properties and the chemical composition of channel layer. The optimized nanocrystalline CuAlO_2_ TFT exhibits a threshold voltage of − 1.3 V, a mobility of ~ 0.1 cm^2^V^−1^ s^−1^, and a current on/off ratio of ~ 10^3^. Compared to vacuum-based magnetron sputtering, our work demonstrates a low-cost, solution-processed CuAlO_2_ TFTs, which represents an important advancement towards the development of complementary metal oxide semiconductor logic circuits.

## Abbreviations

AFM: Atomic force microscopy
*C*_ox_: The areal capacitance of the gate insulator
*I*_on_/*I*_off_: On/off current ratio
*k*: The Boltzmann constant
*L*: The channel length
*N*_t_: The interface trap density
*q*: The elementary charge of electron
SEM: Scanning electron microscopy
SS: The subthreshold slope
*T*: The absolute temperature
TFTs: Thin-film transistors
*V*_T_: Threshold voltage
*W*: The channel width
XPS: X-ray photoelectron spectroscopy
XRD: X-ray diffraction
*μ*_FE_: Filed effect mobility
